# The receptor for advanced glycation end products in ventilator-induced lung injury

**DOI:** 10.1186/s40635-014-0022-1

**Published:** 2014-08-02

**Authors:** Maria T Kuipers, Hamid Aslami, Pieter Roel Tuinman, Anita M Tuip-de Boer, Geartsje Jongsma, Koenraad F van der Sluijs, Goda Choi, Esther K Wolthuis, Joris JTH Roelofs, Paul Bresser, Marcus J Schultz, Tom van der Poll, Catharina W Wieland

**Affiliations:** Laboratory of Experimental Intensive Care and Anesthesiology (L.E.I.C.A), Academic Medical Centre, University of Amsterdam, room M0-220, Meibergdreef 9, Amsterdam, 1105 AZ, The Netherlands; Centre of Experimental and Molecular Medicine, Academic Medical Centre, University of Amsterdam, Amsterdam, 1105 AZ The Netherlands; Department of Intensive Care, Academic Medical Centre, University of Amsterdam, Amsterdam, 1105 AZ The Netherlands; Department of Internal Medicine, Academic Medical Centre, University of Amsterdam, Amsterdam, 1105 AZ, The Netherlands; Department of Anesthesiology, Academic Medical Centre, University of Amsterdam, Amsterdam, 1105 AZ The Netherlands; Department of Pathology, Academic Medical Centre, University of Amsterdam, Amsterdam, 1105 AZ The Netherlands; Department of Respiratory Medicine, Onze Lieve Vrouwe Gasthuis, Amsterdam, 1090 HM The Netherlands; Division of Infectious Diseases, Academic Medical Centre, University of Amsterdam, Amsterdam, 1105 AZ The Netherlands

**Keywords:** Acute lung injury, Mechanical ventilation, Receptor for advanced glycation end products, Inflammation, Innate immunity

## Abstract

**Background:**

Mechanical ventilation (MV) can cause ventilator-induced lung injury (VILI). The innate immune response mediates this iatrogenic inflammatory condition. The receptor for advanced glycation end products (RAGE) is a multiligand receptor that can amplify immune and inflammatory responses. We hypothesized that RAGE signaling contributes to the pro-inflammatory state induced by MV.

**Methods:**

RAGE expression was analyzed in lung brush and lavage cells obtained from ventilated patients and lung tissue of ventilated mice. Healthy wild-type (WT) and RAGE knockout (KO) mice were ventilated with relatively low (approximately 7.5 ml/kg) or high (approximately 15 ml/kg) tidal volume. Positive end-expiratory pressure was set at 2 cm H_2_O during both MV strategies. Also, WT and RAGE KO mice with lipopolysaccharide (LPS)-induced lung injury were ventilated with the above described ventilation strategies. In separate experiments, the contribution of soluble RAGE, a RAGE isoform that may function as a decoy receptor, in ventilated RAGE KO mice was investigated. Lung wet-to-dry ratio, cell and neutrophil influx, cytokine and chemokine concentrations, total protein levels, soluble RAGE, and high-mobility group box 1 (HMGB1) presence in lung lavage fluid were analyzed.

**Results:**

MV was associated with increased RAGE mRNA levels in both human lung brush samples and lung tissue of healthy mice. In healthy high tidal volume-ventilated mice, RAGE deficiency limited inflammatory cell influx. Other VILI parameters were not affected. In our second set of experiments where we compared RAGE KO and WT mice in a 2-hit model, we observed higher pulmonary cytokine and chemokine levels in RAGE KO mice undergoing LPS/high tidal volume MV as compared to WT mice. Third, in WT mice undergoing the LPS/high tidal volume MV, we observed HMGB1 presence in lung lavage fluid. Moreover, MV increased levels of soluble RAGE in lung lavage fluid, with the highest levels found in LPS/high tidal volume-ventilated mice. Administration of soluble RAGE to LPS/high tidal volume-ventilated RAGE KO mice attenuated the production of inflammatory mediators.

**Conclusions:**

RAGE was not a crucial contributor to the pro-inflammatory state induced by MV. However, the presence of sRAGE limited the production of pro-inflammatory mediators in our 2-hit model of LPS and high tidal volume MV.

**Electronic supplementary material:**

The online version of this article (doi:10.1186/s40635-014-0022-1) contains supplementary material, which is available to authorized users.

## Background

Mechanical ventilation (MV) is a crucial intervention in the management of the critically ill but can aggravate lung injury, also known as ventilator-associated lung injury (VALI) in patients and termed ventilator-induced lung injury (VILI) in animal models [1–5]. The exact molecular mechanisms involved in VILI pathogenesis are incompletely understood, but accumulating evidence indicates that MV triggers an inflammatory response in which innate immunity plays a central role [6–11].

Pattern recognition receptors (PRRs) are activated by bacterial products and by damage-associated molecular patterns (DAMPs), which are endogenous molecules released during tissue injury [12]. DAMPs are present in the alveolar compartment during (injurious) MV indicating the potential significance of the DAMP/receptor axis in VILI [7]. The role of some PRRs in VILI has been investigated: VILI is in part mediated by toll-like receptor (TLR) 4 signaling [9,10]. More recently, the importance of the inflammasome pathway was identified [6,8]. Less is known about the role of the receptor for advanced glycation end products (RAGE) in VILI.

RAGE is highly expressed in the lungs, primarily on the basolateral membrane of alveolar type I cells [13] and recognizes a variety of molecules including alarmins such as S100 proteins and high-mobility group box 1 (HMGB1) [14,15]. Soluble RAGE (sRAGE) is a RAGE isoform that lacks the transmembrane and cytosolic part. sRAGE levels can be used as biomarker for alveolar epithelial type I cell injury [16,17]. sRAGE itself may also influence inflammation as it can compete with cell-surface RAGE for ligand engagement [17]. Studies indicated that RAGE ligands are present in the pulmonary compartment during MV: (1) Long-term MV in patients without acute lung injury increased HMGB1 levels in bronchoalveolar lavage fluid (BALF) [18], (2) 4 h of injurious MV in rabbits induced a fivefold increase of HMGB1 levels in BALF and blocking HMGB1 attenuated VILI [19], and (3) S100A12 and S100A8/A9, members of the S100 family of proteins, are found in BALF of patients with acute respiratory distress syndrome (ARDS) [20,21]. RAGE-ligand interaction activates intracellular pathways and induces pro-inflammatory cytokines, proteases, and oxidative stress [17].

We hypothesized that RAGE signaling contributes to the pro-inflammatory state induced by MV. For this, we analyzed the expression of RAGE mRNA in lung brush cells and BALF cells obtained from ventilated patients. In addition, we ventilated wild-type (WT) and RAGE knockout (KO) mice, healthy and with lipopolysaccharide (LPS)-induced lung injury, to study the role of RAGE in VILI. Furthermore, the contribution of soluble RAGE was investigated. To establish the presence of VILI, we analyzed alveolar capillary permeability and the pulmonary inflammatory response.

## Methods

A more detailed description of the methods is provided in Additional file 1.

### Patients

Samples obtained from a previous trial in which patients were randomized to two ventilation strategies during elective surgery were used [22]. The Medical Ethics Committee of the University of Amsterdam approved the study protocol, and informed consent was obtained from all patients [22]. mRNA expression levels of RAGE and hypoxanthine-guanine phosphoribosyl transferase (HPRT) in lung brush samples and BALF cells were determined. Analysis included samples in which RAGE and HPRT were both measurable at both time points allowing paired measurements. The previous trial was not powered to find differences in mRNA levels between the two ventilation groups. We therefore combined the samples from both ventilation strategies.

### Mice

The Animal Care and Use Committee of the Academic Medical Center approved all experiments. Eight- to ten-week-old male RAGE KO mice were generated as described previously [23], backcrossed ten times to a C57Bl/6 background, and bred in the animal facility of the Academic Medical Center (Amsterdam, the Netherlands). C57BL/6 age matched WT mice were purchased from Harlan Laboratories B.V. (Horst, the Netherlands).

#### Design

To obtain a first insight into the role of RAGE in MV-induced inflammation and injury in lungs without pre-existing injury, we randomized healthy WT and RAGE KO mice to 5 h of MV or to a non-ventilated control group. Mice of the MV group were pressure-controlled ventilated with either an inspiratory pressure of 10 cm H_2_O (*V*_T_ ~ 7.5 ml/kg) (LV_T_) or an inspiratory pressure of 18 cm H_2_O (*V*_T_ ~ 15 ml/kg) (HV_T_) (*n* = 6 to 9/group). Respiratory rate was set at 110 breaths/min in the LV_T_ group and 70 breaths/min in the HV_T_ group, positive end-expiratory pressure was set at 2 cm H_2_O during both MV strategies. The physiological characteristics of the VILI model used were published in detail previously [24].

Since it has been shown that lungs with pre-existing injury are more susceptible to the effects of MV [25,26], we randomized in a second set of experiments WT and KO mice with pre-injured lungs (induced by inhalation of 5 μg LPS (*Escherichia coli* L4130, Sigma Aldrich, St. Louis, MO, USA) 1 h before randomization) to the above described ventilation strategies or spontaneously breathing for 5 h.

In a third set of experiments, we analyzed the presence of sRAGE and HMGB1 in our VILI models. In addition, KO mice with LPS-induced lung injury received 50 μg murine sRAGE or vehicle (saline) intratracheally at the start of HV_T_ MV. Recombinant murine his-tagged sRAGE was a kind gift from P. Nawroth. Sample harvesting and processing were done as described previously [8,24].

#### Assays

Total protein was determined using Bradford Protein Assay Kit (OZ Biosciences, Marseille, France). Interleukin (IL)-6, keratinocyte-derived chemokine (KC), macrophage inflammatory protein (MIP)-2, IL-1β, and tumor necrosis factor (TNF)-α levels were measured by enzyme-linked immunosorbent assay (ELISA) (R&D Systems Inc., Minneapolis, MN, USA). sRAGE was measured by Mouse RAGE Duo set ELISA (R&D Systems Inc.) as described before [27]. HMGB1 levels were analyzed by Western blot. Proteins were separated using polyacrylamide gel electrophoresis (Criterion Bis-Tris Precast Gel, Carlsbad, CA, USA); to detect HMGB1, a rabbit polyclonal antibody was used (Abcam Biochemicals, Cambridge, UK). Lung tissue homogenate was used to analyze RAGE and HPRT mRNA levels. Methods were used as described previously [8]. RAGE was stained on paraffin-embedded lung tissue. For this, we used goat anti-mouse RAGE polyclonal antibodies (Neuromics, Edina, MN, USA). Alveolar and bronchial epithelium and vascular endothelium were scored for RAGE presence on a scale 0 (negative) to 3 (very intense); total RAGE staining score represents the sum of all scores.

### Statistical analysis

Data represent mean ± SEM. Human samples were analyzed by paired *t* test or Wilcoxon signed-rank test. One-way analysis of variance with Bonferroni or a Kruskall-Wallis test with Mann-Whitney *U* as *post hoc* analysis was used to analyze multiple groups. To compare two groups, a *t* test or Mann-Whitney *U* test was used. *p* < 0.05 was considered statistically significant.

## Results

### RAGE expression in human lung brush cells is enhanced during MV

**Figure 1:**
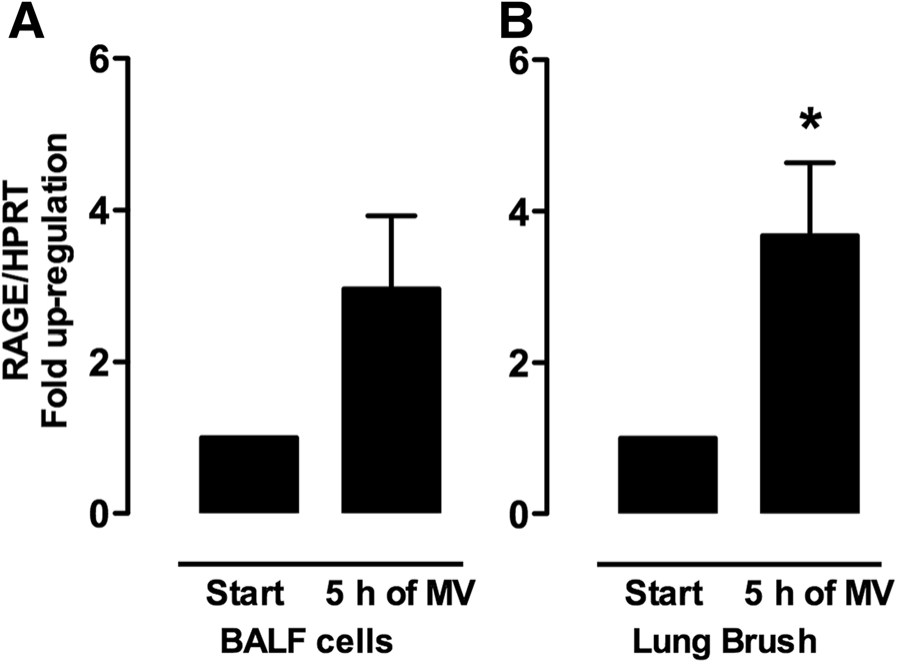
**RAGE gene expression in ventilated patients.** Relative mRNA expression levels of the receptor for advanced glycation end products (RAGE) in human bronchoalveolar lavage fluid cells **(A)** (*n* = 31 pairs) and lung brush samples **(B)** (*n* = 15 pairs). Samples were obtained from patients at baseline and after 5 h of mechanical ventilation (MV). Gene expression was normalized to the house-keeping gene hypoxanthine-guanine phosphoribosyl transferase (HPRT). Data represent mean (SEM), *p < 0.05.

Baseline characteristics, peri-operative parameters and characteristics of the patients included in this study were described in detail previously [22]. BALF cells consisted for more than 99% of macrophages. RAGE mRNA expression levels in these cells (*n* = 31 pairs) tended to be higher after 5 h of MV, but this did not reach statistical significance (*p* = 0.12) (Figure 1). In lung brush samples, RAGE mRNA levels were increased after 5 h of MV (*n* = 15 pairs) (Figure 1). In separate analysis of the two ventilation strategies, no significant differences were found.

### MV enhanced RAGE expression in healthy murine lungs

**Figure 2:**
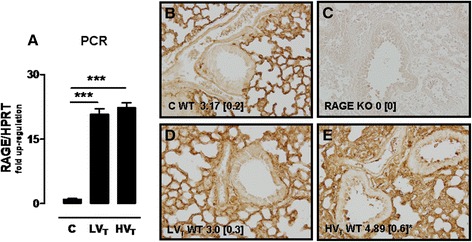
**RAGE expression in 1-hit VILI.** Expression of the receptor of advanced glycation end products (RAGE) in wild-type mice ventilated for 5 h with low tidal volumes (LV_T_ ~ 7.5 ml/kg) or high tidal volumes (HV_T_ ~ 15 ml/kg). Non-ventilated mice **(C)** served as control. RAGE gene expression was measured in lung homogenates and normalized to the house-keeping gene hypoxanthine-guanine phosphoribosyl transferase (HPRT) (*n* = 5 for controls, *n* = 7 ventilated mice/group) **(A)**. Total scores for RAGE expression by immunohistochemical staining of lung tissue (*n* = 6 for controls, *n* = 9 ventilated mice/group) **(B to E)**. Representative view of a lung from non-ventilated control mouse (B), absence of RAGE positivity in the lung of a RAGE KO mouse (C), the lung of a LV_T_-ventilated mouse (D), and increased RAGE expression in the lung of a HV_T_-ventilated mouse (E). Data represent mean (SEM), *p < 0.05, ***p < 0.001 vs LV_T_ and C, staining magnification ×200.

To study if MV in healthy mice increases pulmonary RAGE as well, we determined relative RAGE mRNA expression levels in lung tissue homogenates. Both LV_T_ and HV_T_ ventilation strategies significantly increased RAGE mRNA expression in lung tissue when compared to non-ventilated controls (Figure 2). In addition, we analyzed the expression of RAGE on lung immunohistochemical stainings. Whereas RAGE KO lungs did not show any positive staining, healthy non-ventilated control mice abundantly expressed RAGE in their lungs, predominantly in the alveolar epithelium (Figure 2). The total score for RAGE staining was not affected by LV_T_ ventilation but increased in HV_T_ MV. This was mainly due to *de novo* expression of RAGE on bronchial epithelial cells and endothelium.

### Neutrophil influx into the alveolar compartment is attenuated in RAGE KO mice in 1-hit VILI

**Figure 3:**
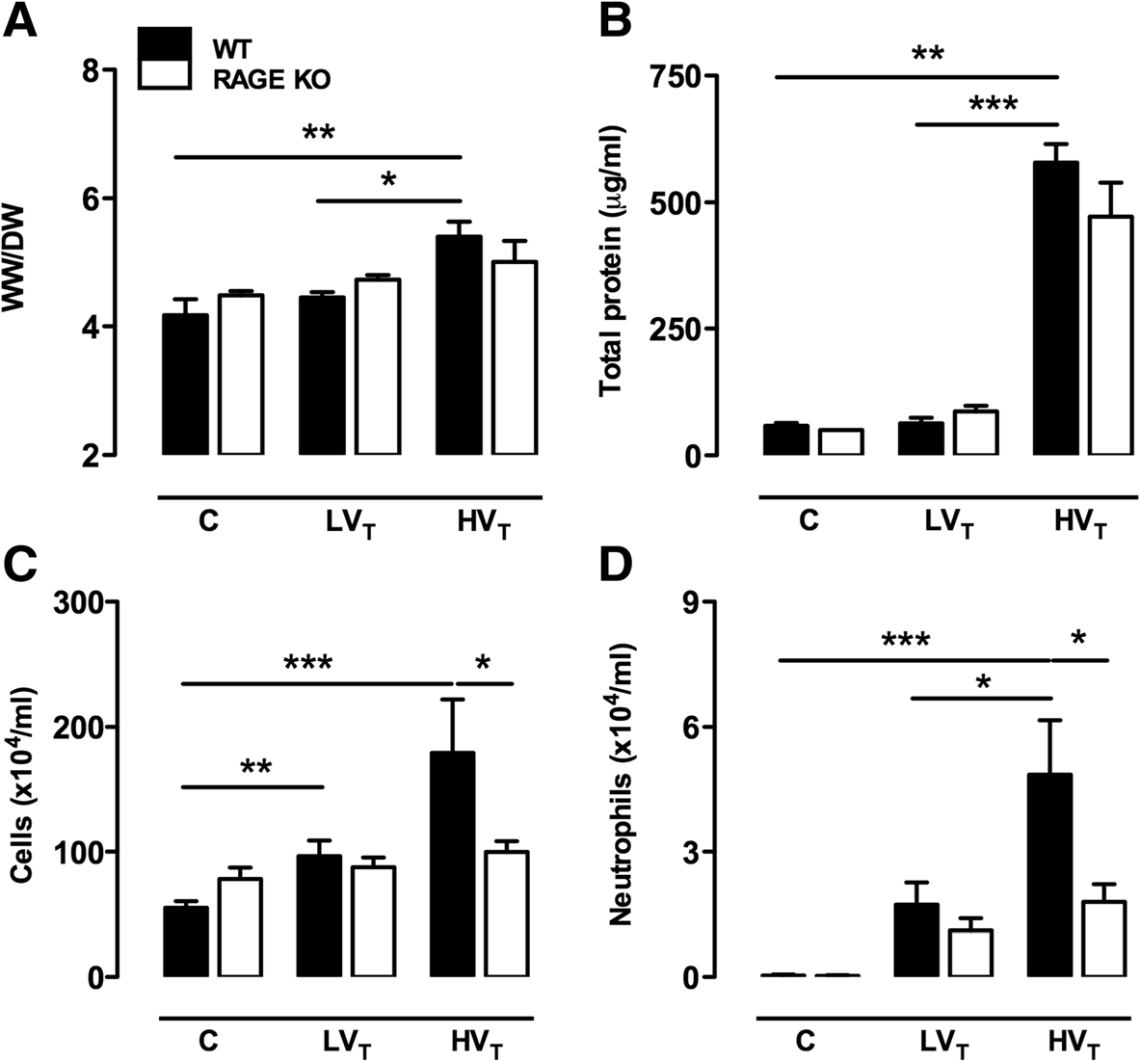
**RAGE in 1-hit VILI.** Lung wet-to-dry ratios **(A)** and total protein level **(B)**, cell influx **(C)**, and neutrophil counts **(D)** in bronchoalveolar lavage fluid of wild-type (WT) and RAGE knockout (KO) mice with healthy lungs ventilated for 5 h with low tidal volumes (LV_T_ ~ 7.5 ml/kg) or high tidal volumes (HV_T_ ~ 15 ml/kg). Non-ventilated mice (C) served as control. Data represent mean (SEM) of *n* = 6 to 9 mice/group. *p < 0.05, **p < 0.01, and ***p < 0.001.

In our 1-hit VILI model, we observed that WT mice subjected to HV_T_ MV demonstrated a marked increase in lung wet-to-dry ratio and total protein level in BALF as compared controls (Figure 3). Further BALF examination demonstrated that both ventilation strategies induced cell influx into the alveolar compartment, with higher levels in HV_T_-ventilated mice. Differential cell counts revealed an increased number of neutrophils, reaching statistical significance for HV_T_-ventilated mice. In addition, HV_T_ MV significantly increased the levels of IL-6, KC, and IL-1β. LV_T_ MV elevated the levels of IL-1β and MIP-2 (Table 1). TNF-α was undetectable. When comparing RAGE KO and WT mice, we observed no significant differences regarding alveolar barrier dysfunction. However, RAGE KO mice demonstrated a significantly lower total cell count and neutrophil influx in BALF after 5 h of HV_T_ MV as compared to WT mice (Figure 3). No differences in concentrations of these inflammatory mediators were found between RAGE KO and WT mice (Table 1).

**Table 1:** **Cytokines and chemokines in bronchoalveolar lavage fluid in 1-hit VILI**

	**C WT**	**C KO**	**LV**_**T**_ **WT**	**LV**_**T**_ **KO**	**HV**_**T**_ **WT**	**HV**_**T**_ **KO**
IL-6 [ng/ml]	B.D.	B.D.	104.0 (17.0)	101.0 (12.4)	156.1 (15.9)***	142.7 (18.6)
KC [ng/ml]	51.4 (0.7)	B.D.	336.7 (70.0)**	463.4 (123.7)	423.4 (94.0)**	505.9 (59.3)
MIP-2 [ng/ml]	B.D.	B.D.	239.6 (56.5)*	233.2 (35.4)	189.2 (18.4)	216.2 (20.6)
IL-1β [ng/ml]	B.D.	B.D.	69.9 (21.9)*	64.9 (15.9)	57.3 (14.5)*	66.0 (21.3)

Cytokines and chemokines [pg/ml] in bronchoalveolar lavage fluid of wild-type (WT) and RAGE knockout (KO) mice with healthy lungs ventilated for 5 h with low tidal volumes (LV_T_ ~ 7.5 ml/kg) or high tidal volumes (HV_T_ ~ 15 ml/kg). Non-ventilated mice (C) served as control. Data represent mean (SEM) of *n* = 6 to 9 mice/group. *p < 0.05; **p < 0.01; ***p < 0.001 vs controls. B.D., below detection.

### RAGE KO mice have elevated levels of pro-inflammatory mediators in 2-hit VILI

**Figure 4:**
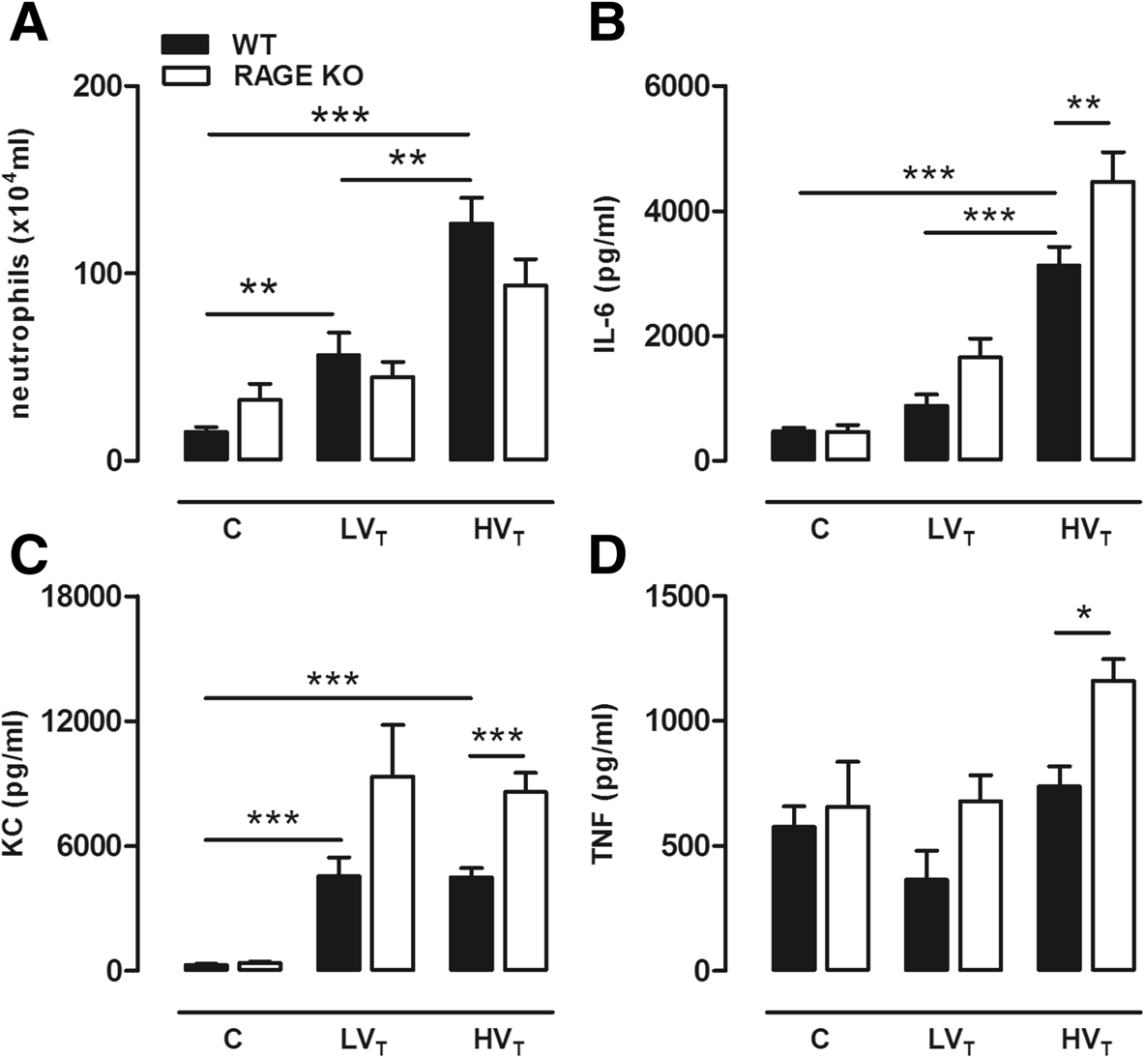
**RAGE in 2-hit VILI.** Neutrophil counts **(A)** and levels of interleukin (IL)-6 **(B)**, keratinocyte-derived chemokine (KC) **(C)** and tumor necrosis factor (TNF)-α **(D)** in bronchoalveolar lavage fluid of wild-type (WT) and RAGE knockout (KO) mice in a 2-hit lung injury model of lipopolysaccharide (LPS) exposure followed by mechanical ventilation for 5 h with low tidal volumes (LV_T_ ~ 7.5 ml/kg) or high tidal volumes (HV_T_ ~ 15 ml/kg). LPS-exposed non-ventilated mice (C) served as control. Data represent mean (SEM) of *n* = 6 to 9 mice/group. *p < 0.05, **p < 0.01, and ***p < 0.001.

MV enhanced LPS-induced lung injury in our 2-hit VILI model, most clearly demonstrated in HV_T_-ventilated mice (Figure 4). When comparing RAGE KO and WT mice, we observed that RAGE deficiency did not affect lung wet-to-dry ratio, total protein level, and cell influx in LPS-exposed and LPS/MV-treated groups (Additional file 2). Also, neutrophil influx was not significantly different between WT and RAGE KO mice (Figure 4). Remarkably, in our 2-hit setting, RAGE KO mice demonstrated elevated cytokine and chemokine levels in BALF. IL-6, TNF-α, and KC were higher when LPS-induced lung injury was combined with MV, reaching significance for the RAGE KO mice of the LPS/HV_T_ MV group (Figure 4). Of note, MIP-2 and IL-1β levels were not different (Additional file 2).

### sRAGE attenuated inflammation in RAGE KO mice

**Figure 5:**
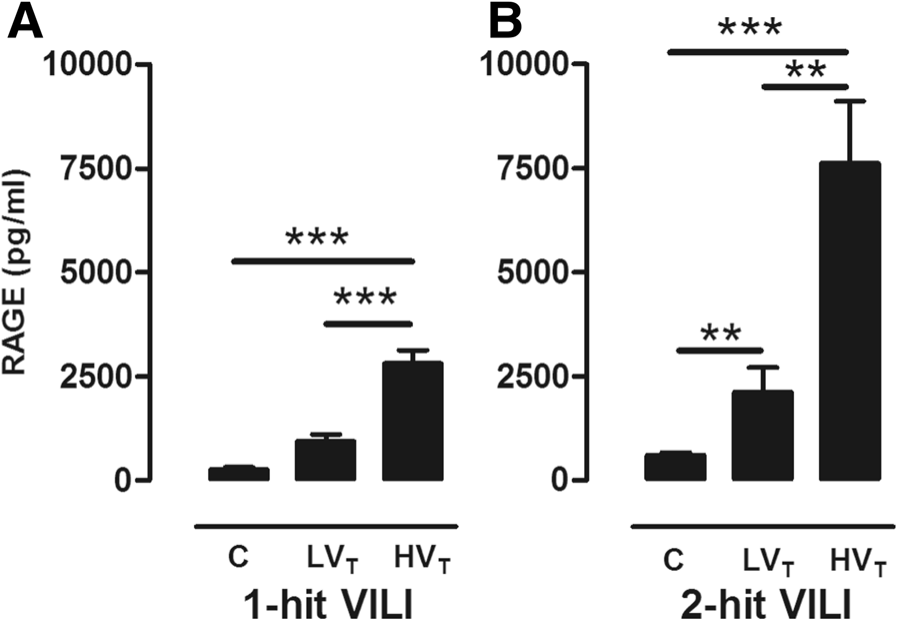
**sRAGE in lung lavage fluid in 1-hit VILI.** Soluble RAGE (sRAGE) levels in bronchoalveolar lavage fluid (BALF) of wild-type (WT) mice ventilated for 5 h with low tidal volumes (LV_T_ ~ 7.5 ml/kg) or high tidal volumes (HV_T_ ~ 15 ml/kg) (VILI) **(A)**. In addition, sRAGE **(B)** in BALF of WT mice in a 2-hit model of lipopolysaccharide (LPS) exposure followed by mechanical ventilation for 5 h with low tidal volumes (LV_T_ ~ 7.5 ml/kg) or high tidal volumes (HV_T_ ~ 15 ml/kg). Data represent mean (SEM) of *n* = 6 to 9 mice/group. **p < 0.01, and ***p < 0.001.

**Figure 6:**
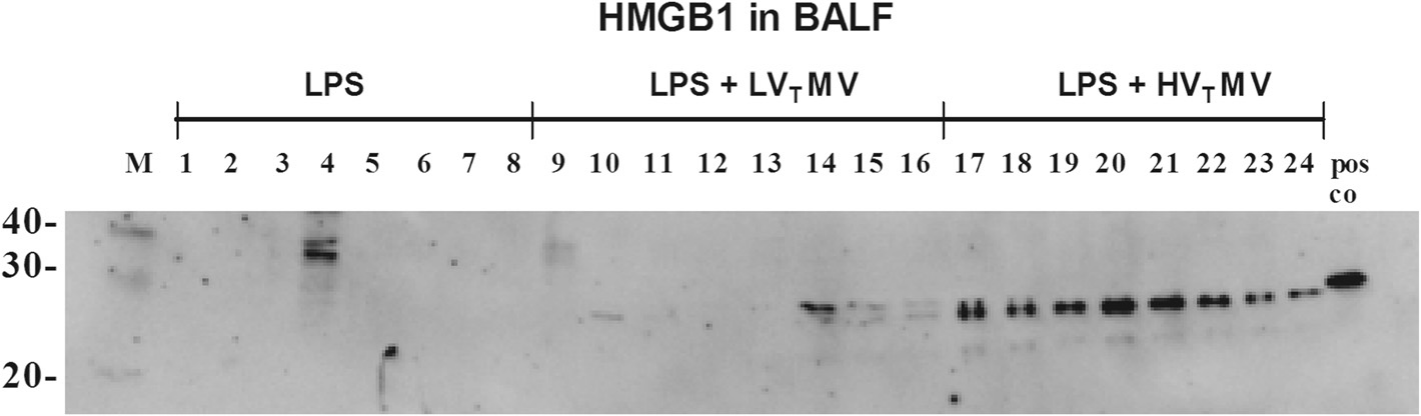
**HMGB1 in lung lavage fluid in 2-hit VILI.** High mobility group box-1 (HMGB1) levels in bronchoalveolar lavage fluid (BALF) of wild-type mice in a 2-hit model of lipopolysaccharide (LPS) exposure followed by spontaneous breathing or mechanical ventilation for 5 h with low tidal volumes (LV_T_ ~ 7.5 ml/kg) or high tidal volumes (HV_T_ ~ 15 ml/kg), *n* = 8 mice/group.

Next, we measured RAGE ligand HMGB1 and sRAGE levels in our VILI models. HV_T_ MV of healthy mice resulted in an increased BALF sRAGE concentration compared to LV_T_ MV and non-ventilated controls (Figure 5). Levels further increased during the LPS/MV double hit in which the highest levels were found in the HV_T_-ventilated group. HMGB1 was undetectable in 1-hit HV_T_-ventilated mice. In our 2-hit model, we observed that in lung lavage of animals with LPS-induced lung injury or LPS-induced injury combined with LV_T_ MV, HMGB1 was barely detectable (Figure 6). However, HMGB1 was clearly present in BALF of mice that underwent the LPS/HV_T_ MV double hit (Figure 6). We next verified whether sRAGE might have protective effects during MV by administering sRAGE (or vehicle) to LPS/HV_T_-ventilated RAGE KO mice. We observed that total protein, cell count, and neutrophil influx were not affected by sRAGE administration (Table 2). However, when analyzing cytokine and chemokine levels in BALF, we found that sRAGE-treated mice displayed significantly lower levels of IL-6, KC, and MIP-2 (Table 2).

**Table 2:** **VILI parameters after sRAGE treatment in RAGE KO**

	**Vehicle i.t.**	**sRAGE i.t.**
Cell count [×10^4^]	112 (1.6)	91 (0.9)
Neutrophil influx [×10^4^]	103 (15)	86 (10)
Total protein [μg/ml]	623 (32)	614 (44)
IL-6 [pg/ml]	3162 (291)	1875 (243)**
KC [pg/ml]	8831 (876)	6016 (867)*
MIP-2 [pg/ml]	6115 (688)	2827 (379)***
IL-1β [pg/ml]	676 (69)	850 (122)
TNF-α [pg/ml]	1274 (118)	1425 (141)

Cell count, neutrophil count, total protein, and cytokine and chemokine levels in bronchoalveolar lavage fluid of RAGE knockout (KO) mice with LPS-injured lungs treated with soluble RAGE or vehicle at the start of 5 h of high tidal volume (HV_T_ ~ 15 ml/kg) mechanical ventilation. Data represent mean (SEM) of *n* = 7 to 8 mice/group. *p < 0.05; **p < 0.01; ***p < 0.001. i.t., intra tracheal.

## Discussion

We demonstrate the following: (1) RAGE expression is up-regulated during 5 h of MV in human lung brush cells and in murine lungs. (2) RAGE contributes to inflammatory cell influx in 1-hit VILI, but it does not affect other VILI parameters. (3) In a 2-hit VILI setting, RAGE deficiency is not protective; on the contrary, the lack of RAGE resulted in an enhanced inflammatory response. (4) sRAGE administration to RAGE KO mice undergoing the LPS/HV_T_ MV double hit in part reverses this phenotype.

This is the first study that investigated the role of RAGE in the development of MV-induced inflammation and injury. Others have studied RAGE in other lung injury models such as lung fibrosis, hyperoxyia, LPS, and infection [28–31]. In line with previous studies, we observed that in healthy lungs RAGE is already abundantly expressed [16,28,29]. Our finding that MV increases RAGE expression in mouse lungs extends a previous investigation studying RAGE in hyperoxia-induced lung inflammation: 4 days of hyperoxia elevated pulmonary RAGE as demonstrated by immunostaining, immunoblotting, and real-time polymerase chain reaction (PCR) [29]. In contrast, other sterile lung inflammatory disorders reported no impact on pulmonary RAGE expression (LPS instillation) [16] or reported reduced RAGE levels (in pulmonary fibrosis models) [28,30]. This might indicate that the presence of a sustained pro-inflammatory stimulus such as hyperoxia and MV has more impact on RAGE expression. In line, also lung infection models, with the continuous presence of bacteria, reported RAGE up-regulation [31].

In our 1-hit VILI model, RAGE deficiency was associated with a reduced total cell and neutrophil influx into the alveolar compartment. RAGE activation may enhance neutrophil migration by increasing the release of inflammatory mediators. However, we observed no differences in BALF cytokine and chemokine levels at the time point investigated. RAGE itself has also been implicated in the regulation of cell migration: it can function as a counter receptor for leukocyte integrins [32]. *In vitro* studies showed that RAGE can bind the β2 integrin MAC-1 and p150,95 [32]. From these findings, it can be speculated that RAGE-dependent neutrophil adhesion also contributed in our 1-hit VILI model.

Our current analysis revealed no differences in inflammation in pulmonary LPS-exposed RAGE KO and WT mice. These results are in line with a previous report also showing a similar degree of inflammation between RAGE-deficient and WT mice, here 24 h after intratracheal LPS challenge [33]. However, a recent *in vitro* study reported evidence for RAGE-LPS interaction [34]. This group also demonstrated that RAGE contributes to LPS-induced nuclear factor-κB activation in isolated peritoneal macrophages, while *in vivo* RAGE KO mice displayed reduced mortality and inflammation after intraperitoneal LPS-induced shock [34]. As such, the importance of the RAGE-LPS interaction seems to vary in different organ tissues; in the lungs, RAGE-LPS signaling appears less important.

RAGE KO mice undergoing the LPS/HV_T_ MV double hit were not protected from VILI. Instead, they displayed more lung inflammation. This is a remarkable finding since previous findings demonstrated that RAGE deficiency led to attenuated inflammation in sterile lung injury models such as bleomycin-induced and hyperoxia-induced lung injury [29,35]. However, inflammation was evaluated at a much later time point in these studies: several days after the start of the experiment. Possibly, deficiency of RAGE in our MV model might have influenced respiratory mechanics: pulmonary RAGE is also important for adherence of epithelial cells towards the basal membrane [36]. However, a previous study reported no differences in airway and tissue resistance, compliance, and elastance between healthy RAGE KO and WT mice [37]. We repeated our LPS/HV_T_ MV double hit group in a MV model with volume-controlled ventilation, to exclude possible interference of lung mechanics. Again, an increased inflammatory response in RAGE KO mice was observed (data not shown).

Another explanation for our finding is that the lack of sRAGE was unfavorable for the inflammatory response since sRAGE can scavenge ligands that also have the potential to activate other PRRs. HMGB1 for example can activate not only RAGE but also TLR4 [38]. Moreover, TLR4 signaling clearly contributes to VILI development [9,10,39]. We observed elevated HMGB1 levels in mice undergoing the LPS/HV_T_ MV double hit, extending a previous study reporting increased HMGB1 levels after 4 h of extremely high tidal volume (30 ml/kg) MV [19]. Blocking HMGB1 in that study improved alveolar barrier dysfunction and limited neutrophil influx and TNF-α release. In addition, S100A8/A8 proteins, which are as well present in our murine VILI model, also have the potential to trigger both RAGE and TLR4 [15,40]. The observation that sRAGE administration to RAGE KO mice in part reversed the increased inflammation present in RAGE KO mice indeed suggests that certain ligands were sequestered by sRAGE that would have interacted with other PRRs in the absence of sRAGE. If sRAGE administration could also have therapeutic potential in WT mice in our VILI model is an interesting question for future research. However, a recent study demonstrated that neither intratracheal nor intraperitoneal sRAGE treatment affected LPS-induced or *E. coli*-induced acute pulmonary inflammation, even though HMGB1 was also increased in these models [33]. In contrast, intraperitoneal sRAGE attenuated lung injury in systemically LPS-challenged mice [41].

We chose to ventilate our mice with low or high tidal volume MV as both strategies may reveal relevant information for clinical practice. Low tidal volume MV is widely practiced since the ARDS network group convincingly demonstrated that this reduces morbidity and mortality in ARDS [2]. However, ARDS is a very heterogeneous disease: some lung regions are poorly aerated placing other healthier lung regions at risk for overinflation [42]. It has been shown that even with the use of lung protective ventilator settings one third of the ARDS patients still experience regional tidal hyperinflation [42]. We therefore believe it is still an important translational effort to ventilate animals with higher tidal volumes. In addition, a pulmonary pro-inflammatory state makes the lung more vulnerable to a second hit such as MV [25,26]. Therefore, to mimic ventilation in the presence of pulmonary co-morbidities, we added an additional injurious stimulus to our MV model. Nonetheless, our 1-hit VILI model is still important to give insights into RAGE signaling in ventilated healthy lungs.

Our study has several limitations. First, we used patient samples from a previous study in which the effect of two ventilation strategies on inflammation and coagulation was analyzed. This study was, however, not powered to find differences in mRNA levels between the two groups. We therefore combined the samples from both ventilation strategies. Second, we used a short-term VILI model to analyze the role of RAGE. Although short-term MV models are commonly used [8–11,24–26,39], as it is technically very difficult to ventilate rodents for days, a long-term MV model would make the translation of results to clinical practice easier. Third, the tracheotomy and the use of anesthesia might also have influenced inflammation in all ventilated animals. It would be ideal to have a control group of anesthetized, tracheotomized spontaneously breathing mice. Unfortunately, this results in hypoventilation, respiratory acidosis, and death.

## Conclusions

In conclusion, our data indicate that RAGE is not a crucial pro-inflammatory receptor in the development of MV-induced inflammation. However, the presence of sRAGE limited the production of pro-inflammatory mediators in ventilated diseased RAGE KO lungs. Further research is needed to study possible therapeutic potential of sRAGE in VILI.

## Additional files

Additional file 1:
**A detailed describtion of the methods.**
Additional file 2:
**RAGE in 2 hit VILI.**

## Abbreviations

ARDS: acute respiratory distress syndrome
BALF: bronchoalveolar lavage fluid
DAMPs: damage-associated molecular patterns
ELISA: enzyme-linked immunosorbent assay
HMGB1: high-mobility group box 1
HPRT: hypoxanthine-guanine phosphoribosyl transferase
IL: interleukin
KC: keratinocyte-derived chemokine
KO: knockout
LPS: lipopolysaccharide
MIP: macrophage inflammatory protein
MV: mechanical ventilation
PEEP: positive end-expiratory pressure
PRRs: pattern recognition receptors
RAGE: receptor for advanced glycation end products
sRAGE: soluble RAGE
TLR: toll-like receptor
TNF: tumor necrosis factor
VILI: ventilator-induced lung injury
WT: wild type
