# Supplementary material for: The receptor for advanced glycation end products in ventilator-induced lung injury
Source: Intensive Care Med Exp. 2014 Aug 2;2:22. doi: 10.1186/s40635-014-0022-1 (PMC4678142; doi:10.1186/s40635-014-0022-1)
Supplement: Additional file 1: — A detailed describtion of the methods. [file 40635_2014_22_MOESM1_ESM.doc]

**Online data supplement 1**

**Materials and Methods**

**Patients**

Bronchial brush samples and bronchoalveolar lavage fluid (BALF) cells from a previously published randomized controlled trial in which patients without pre-existing lung injury were mechanically ventilated for 5 hours during elective surgery were used (1). The Medical Ethics Committee of the University of Amsterdam approved the study protocol and informed consent was obtained from all patients. Adult patients scheduled for a surgical procedure with an estimated duration of 5 hours or longer were eligible for this study. Exclusion criteria were: history of any lung disease, use of immunosuppressive medication, recent infections, previous thromboembolic disease and recent ventilatory support.

*Study design*

Study design was described in detail previously (1). Patients without lung injury scheduled for elective surgery were randomized to mechanical ventilation (MV) with either a conventional tidal volume (V_T_) of 12 ml/kg ideal body weight and 0 cm H_2_O positive end expiratory pressure (PEEP) or 6 ml/kg and 10 cm H_2_O PEEP. The inspired oxygen fraction was 0.4, the inspiration-to-expiration ratio 1:2, and the respiratory rate was adjusted to achieve normocapnia. Bronchial brushes and BALF were collected during bronchoscopy which was performed twice in each patient: the first directly after induction of anaesthesia and start of MV in the right middle lobe or lingula, and the second was performed in the contralateral lung 5 hours thereafter, either perioperatively or directly postoperatively. BALF was processed as previously described (1). BALF cells were resuspended in ice cold phosphate buffered saline and were partially used for absolute and differential cell counts using Giemsa-stained cytospin preparations. The remaining cells were dissolved in RNAlater (Ambion, Austin, TX) and stored at -80 ˚C. Bronchial brushes were directly placed into RNAlater. After several minutes and thorough vortexing, the brush was removed. RNA was purified using RNeasy mini kit system (Qiagen, Valencia, CA) according to the manufacturer’s protocol. Lung brush samples and BALF cells were used to determine mRNA expression levels of receptor for advanced glycation end-products (RAGE) and hypoxanthine-guanine phosphoribosyl transferase (HPRT). Analysis only included patient samples in which both RAGE and HPRT were measurable at both time points allowing paired measurements, the data from both ventilation strategies was combined.

**Mice**

The Animal Care and Use Committee of the Academic Medical Center of the University of Amsterdam approved all experiments. Animal procedures were carried out in compliance with Institutional Standards for Human Care and Use of Laboratory Animals.

Eight- to 10 week old male RAGE knockout (KO) mice were generated as described previously (2), backcrossed 10 times to a C57Bl/6 background, and bred in the animal facility of the Academic Medical Center (Amsterdam, the Netherlands). C57BL/6 age matched wild-type (WT) mice were purchased from Harlan laboratories B.V. (Horst, the Netherlands).

*Experimental groups*

WT and RAGE KO mice were randomly assigned to 2 different ventilation strategies (see below, MV strategies) or to a non-ventilated control group. Two sets of experiments were performed with 6-9 mice per group. In the first set of experiments healthy RAGE KO and WT mice were randomized. After 5 hours mice were sacrificed and right lungs were used to collect BALF and left lungs for wet/dry ratio. An additional group of WT mice were ventilated to obtain lungs for RAGE lung tissue staining and mRNA analysis. In the second set of experiments we used WT and RAGE KO mice with pre-injured lungs induced by 5 μg LPS (*E. coli* L4130, Sigma Aldrich) intranasally under 2-3% isoflurane inhalation anesthesia. After 1 hour mice were randomized to a ventilation strategy, again for 5 hours. The right lungs was used to collect BALF and left lungs for wet/dry ratio. To study the importance of sRAGE in the 2-hit setting a third experiment was performed (n=8 mice/group). Here, RAGE KO mice with LPS-induced lung injury received 50μg murine sRAGE or vehicle (saline) intratracheal at start of 5 hours of high tidal volume MV. Recombinant murine his-tagged sRAGE was a kind gift from P. Nawroth.

*Instrumentation and anesthesia*

Methods used in this VILI model were published in detail previously (3;4). In short, mice received an intraperitoneal bolus of 1 ml normal saline 1 hour before start of anesthesia and initiation of MV. In ventilated mice, a tracheotomy was performed and an Y–tube connector was inserted into the trachea under general anesthesia with an intraperitoneal injection of 0.75 μl/gram body weight of 1.26 ml 100 mg/ml ketamine, 0.2 ml 1 mg/ml dexmedetomidine, and 1 ml 0.5 mg/ml atropine in 5 ml normal saline. Maintenance anesthesia consisted of 1 μl per/gram body weight of 0.72 ml 100 mg/ml ketamine, 0.08 ml 1 mg/ml dexmedetomidine, and 0.3 ml 0.5 mg/ml atropine in 20 ml normal saline. Maintenance mix was administered hourly via an intraperitoneal catheter, every 30 minutes 0.2 ml sodium bicarbonate (200 mmol/l NaHCO_3_) was administered via the same catheter. Throughout the experiments rectal temperature was maintained between 36.5 – 37.5 ^0^C using a warming pad.

*MV strategies and sampling*

Ventilated animals were placed in a supine position and connected to a human ventilator (Servo 900 C, Siemens, Sweden). Mice were pressure controlled ventilated for 5 hours with either an inspiratory pressure of 10 cm H_2_O (V_T_ ~ 7.5 ml/kg, LV_T_) or an inspiratory pressure of 18 cm H_2_O (V_T_ ~ 15 ml/kg, HV_T_). Respiratory rate was set at 110 breaths/min and 70 breaths/min respectively. PEEP was set at 2 cm H_2_O during both MV strategies. The fraction of inspired oxygen was kept at 0.5 and inspiration to expiration ratio was 1:1. After 5 hours of MV mice were sacrificed by withdrawing blood from the carotid artery. Sample harvesting, processing and cell counts were done as described previously (3;4). For RNA analysis, lungs were homogenized in 4 volumes of saline and 50 µl was transferred in Tripure (Roche, Woerden, the Netherlands).

*Immunostaining of RAGE*

Immunostaining of RAGE was performed on slides after deparaffinization and rehydration. One % H_2_O_2_ in methanol was used to quench endogenous peroxidase activity. Goat anti-mouse RAGE polyclonal antibodies (Neuromics, Edina, MN, USA) were used as primary antibody, biotinylated rabbit anti-goat antibodies (DakoCytomation, Glostrup, Denmark) as secondary antibody. For detection ABC solution (DakoCytomation, Glostrup, Denmark) was used. Visualization was obtained with DAB peroxidase (Sigma, St Louis, MO) as substrate. Lung stainings were scored for RAGE presence on a scale 0 (negative) – 3 (very intense): alveolar and bronchial epithelium and vascular endothelium were analyzed by a pathologist blinded for group identity, total RAGE staining score represents the sum of all scores.

*Assays.*

Total protein levels in BALF were determined using a Bradford Protein Assay Kit (OZ Biosciences, Marseille, France) according to manufacturers’ instructions with a detection limit of 50μg/ml. Interleukin (IL)–6, keratinocyte–derived chemokine (KC), macrophage inflammatory protein (MIP)-2, IL-1β, and tumor necrosis factor (TNF)-α levels were measured by enzyme–linked immunosorbent assay (ELISA) according to the manufacturer’s instructions (R&D Systems Inc., Minneapolis, MN, USA). Detection limits were 51 pg/ml for IL-6, KC, and TNF- α, 153 pg/ml for MIP-2 and 25 pg/ml for IL-1β. sRAGE levels were measured by Mouse RAGE Duo set ELISA (R&D Systems Inc., Minneapolis, MN, USA) as described before with a detection limit of 102pg/ml (5).

*HMGB-1 western blot*

Sixteen μl BALF was separated by polyacrylamide gel electrophoresis (Criterion Bis-Tris Precast Gel, Carlsbad, CA, USA). Proteins were transferred to polyvinylidene membranes, blocked in blocking buffer containing 5% non-fat dry milk proteins with Tween 20 in 50 mM Tris, 150 mM NaCl (pH 7.4, TBS), washed, and incubated overnight with rabbit polyclonal HMGB-1 antibodies (Abcam Biochemicals, Cambridge, UK) at 4˚C. After washing, membranes were probed with peroxidase-labelled Rabbit-IgG1-HRP (Cell Signalling Technology, Danvers, MA) for 1 hour at room temperature in TBS. Again after washing, blots were imaged with Lumi-Light^Plus^ Western Blotting Substrate (Roche, Mijdrecht, the Netherlands).

*mRNA expression analysis*

Complementary DNA synthesis from human and murine RNA was performed by a reverse transcription reaction using Moloney murine leukemia virus reverse transcriptase (Invitrogen) and oligo dT (Invitrogen) as described before (4). Quantitative polymerase chain reactions were done using lightCycler®SYBR green I master mix (Roche, Mijdrecht, the Netherlands) and analyzed in a LightCycler 480 (Roche) apparatus with the following conditions: 5 min 95° C hot-start, followed by 40 cycles of amplification (95° C for 10 seconds, 60° C for 5 seconds, 72° C for 15 seconds). Standard curves were constructed by polymerase chain reactions on serial dilutions of a concentrated complementary DNA sample for quantifications. Data were analyzed using LightCycler software. The following human primer sequences were used: RAGE forward primer 5’-gccagaaggtggagcagtag-3’ and reverse primer 5’-acaagatgaccccaatgagc-3’ HPRT forward primer 5’-tgctgacctgctggattaca-3’ and reverse primer 5’-cctgaccaaggaaagcaaag-3’, Murine primer sequences were: RAGE forward primer 5’-aacccatcctaccttctcctg-3’ and reverse primer 5’-cccaccaggagcgactatt-3’, HPRT forward primer 5’-tcctcctcagaccgctttt-3’ and reverse primer 5’-cctggttcatcatcgctaatc-3’.

**References**

(1) Choi G, Wolthuis EK, Bresser P, Levi M, van der Poll T, Dzoljic M, Vroom MB, Schultz MJ. (2006) Mechanical ventilation with lower tidal volumes and positive end-expiratory pressure prevents alveolar coagulation in patients without lung injury. Anesthesiology 105:689-695.

(2) Liliensiek B, Weigand MA, Bierhaus A, Nicklas W, Kasper M, Hofer S, Plachky J, Grone HJ, Kurschus FC, Schmidt AM, Yan SD, Martin E, Schleicher E, Stern DM, Hammerling GG, Nawroth PP, Arnold B (2004) Receptor for advanced glycation end products (RAGE) regulates sepsis but not the adaptive immune response. J Clin Invest 113;1641-1650.

(3) Wolthuis EK, Vlaar AP, Choi G, Roelofs JJ, Juffermans NP, Schultz MJ (2009) Mechanical ventilation using non-injurious ventilation settings causes lung injury in the absence of pre-existing lung injury in healthy mice. Crit Care 13(1):R1.

(4) Kuipers MT, Aslami H, Janczy JR, van der Sluijs KF, Vlaar AP, Wolthuis EK, Choi G, Roelofs JJ, Flavell RA, Sutterwala FS, Bresser P, Leemans JC, van der Poll T, Schultz MJ, Wieland CW (2012) Ventilator-induced lung injury is mediated by the NLRP3 inflammasome. Anesthesiology 116:1104-1115.

(5) Kalea AZ, Reiniger N, Yang H, Arriero M, Schmidt AM, Hudson BI (2009) Alternative splicing of the murine receptor for advanced glycation end-products (RAGE) gene. FASEB J 23:1766-1774.
