# Supplementary material for: The receptor for advanced glycation end products in ventilator-induced lung injury
Source: Intensive Care Med Exp. 2014 Aug 2;2:22. doi: 10.1186/s40635-014-0022-1 (PMC4678142; doi:10.1186/s40635-014-0022-1)
Supplement: Additional file 2: — RAGE in 2 hit VILI. [file 40635_2014_22_MOESM2_ESM.doc]

**Online data supplement 2**

**RAGE in 2-hit VILI**

**
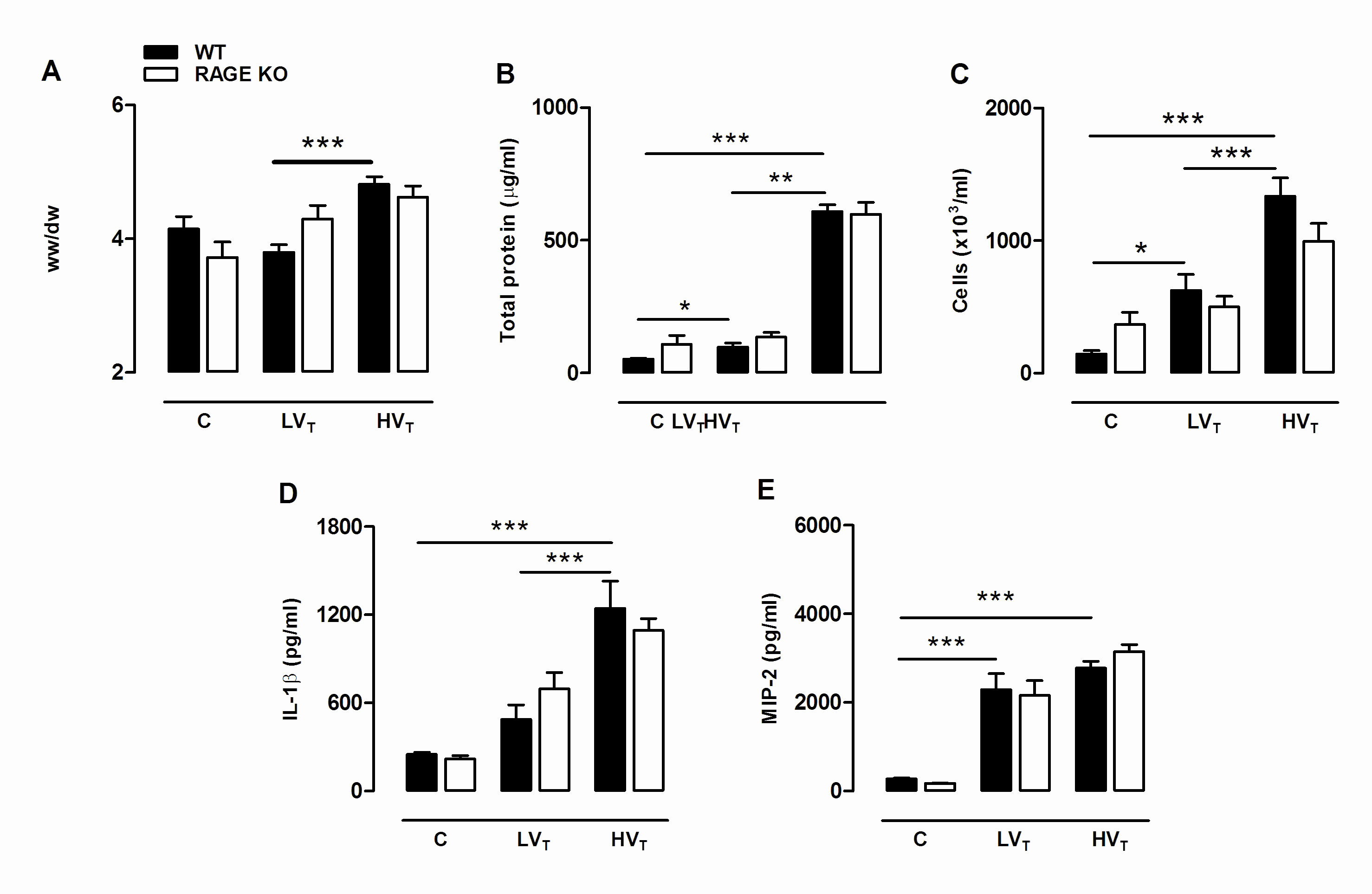
**

Lung wet/dry ratio (**A**), and total protein levels (**B**), cell counts (**C**), interleukin (IL)-1β (**D**) and macrophage-inflammatory protein-2 (MIP-2) (**E**) levels in bronchoalveolar lavage fluid of wild-type (WT) and RAGE knockout (KO) mice in a 2-hit lung injury model of lipopolysaccharide (LPS) exposure followed by mechanical ventilation for 5 hours with low tidal volumes (LV_T_) (~7.5 ml/kg) or high tidal volumes (HV_T_) (~15 ml/kg). LPS-exposed non-ventilated mice (C) served as control. Data represent mean (SEM) of n=6-9 mice/group. *p<0.05, **p<0.01, ***p<0.001.
